# Body Mass Index and Mortality in Chronic Obstructive Pulmonary Disease: A Meta-Analysis

**DOI:** 10.1371/journal.pone.0043892

**Published:** 2012-08-24

**Authors:** Chao Cao, Ran Wang, Jianmiao Wang, Hansvin Bunjhoo, Yongjian Xu, Weining Xiong

**Affiliations:** 1 Department of Respiratory Medicine, Tongji Hospital, Tongji Medical College, Huazhong University of Science and Technology, Wuhan, China; 2 Department of Respiratory Medicine, Affiliated Hospital, Medical College, Ningbo University, Ningbo, China; The University of Edinburgh, United Kingdom

## Abstract

**Background:**

The association between body mass index (BMI) and mortality in patients suffering from chronic obstructive pulmonary disease (COPD) has been a subject of interest for decades. However, the evidence is inadequate to draw robust conclusions because some studies were generally small or with a short follow-up.

**Methods:**

We carried out a search in MEDLINE, Cochrane Central Register of Controlled Trials, and EMBASE database for relevant studies. Relative risks (RRs) with 95% confidence interval (CI) were calculated to assess the association between BMI and mortality in patients with COPD. In addition, a baseline risk-adjusted analysis was performed to investigate the strength of this association.

**Results:**

22 studies comprising 21,150 participants were included in this analysis. Compared with patients having a normal BMI, underweight individuals were associated with higher mortality (RR  = 1.34, 95% CI  = 1.01–1.78), whereas overweight (RR  = 0.47, 95% CI  = 0.33–0.68) and obese (RR  = 0.59, 95% CI  = 0.38–0.91) patients were associated with lower mortality. We further performed a baseline risk-adjusted analysis and obtained statistically similar results.

**Conclusion:**

Our study showed that for patients with COPD being overweight or obese had a protective effect against mortality. However, the relationship between BMI and mortality in different classes of obesity needed further clarification in well-designed clinical studies.

## Introduction

Chronic obstructive pulmonary disease (COPD), a common disease with a high burden on healthcare resources, is predicted to be the third leading cause of death worldwide by the year 2020 [1]. Apart from its effects in the lungs, COPD has been redefined as a systemic disease in recent years due to its significant extrapulmonary manifestations [2]. Although the hallmark feature of COPD is airflow obstruction, it was poorly predicted by Forced Expiratory Volume in the first second (FEV_1_) only [3], [4]. Thus, many other independent predictors of outcomes have been identified including frequency of acute exacerbations and hospital admissions [5], exercise capacity [3], nutritional status [6], depressive symptoms [7], and biomarkers of systemic and bronchial inflammation [8]. Among these, the most extensively studied and conveniently used predictor is the alterations of the Body Mass Index (BMI).

BMI is the weight in kilograms divided by the square of the height in meters. The association between BMI and mortality in patients suffering from COPD has been a subject of interest for decades. Even though weight loss commonly occurs among these patients, it is not clear whether this is an epiphenomenon of severity of the disease or an independent risk factor [9]. Studies with small sample size or a short follow-up appeared to lack sufficient power to detect such an association. In addition, data from some studies were insufficient to adjust the influence of age, gender, smoking, and lung function. To comprehensively synthesize the evidence relating to this issue, we conducted this systematic review to examine if the BMI in patients with COPD is a significant predictor of mortality after being adjusted for other prognostic factors.

## Materials and Methods

### Identification and eligibility of relevant studies

We carried out a search in MEDLINE, Cochrane Central Register of Controlled Trials (CENTRAL), and EMBASE databases covering all papers published till March 2011, using the following search terms: *body mass index, chronic obstructive pulmonary disease, and cohort studies*, as well as combinations of these terms. We retrieved all eligible studies, checked their bibliographies for other relevant publications, and examined the abstracts of relevant scientific meetings to ensure complete review of available studies. We also made efforts to contact the author when relevant data were unclear.

### Study selection

Individual studies had to meet the following criteria to be included: (1) study population originating from a well-established general cohort; (2) diagnosis of COPD according to pulmonary function test; (3) mortality reported in COPD patients according to BMI category; (4) published in English; (5) the availability of data for analysis; and (6) BMI categorized into four groups according to World Health Organization (WHO) guidelines (http://apps.who.int/bmi/index.jsp?introPage=intro_3.html): (a) underweight (≤18.5 kg/m^2^), (b) normal weight (18.5–24.9 kg/m^2^), (c) overweight (25.0–29.9 kg/m^2^), and (d) obese (≥30.0 kg/m^2^) [10]. We anticipated all the included studies to be according to WHO BMI classification guidelines. However, the cutoff point for underweight patients was controversial, and 20 kg/m^2^ or 21 kg/m^2^ was also accepted. Major exclusion criteria of studies were if (1) the BMI classification was not according to WHO guidelines or (2) the participant was without COPD at baseline. The study quality was assessed using the 9-star Newcastle-Ottawa Scale [11].

### Data extraction

Two investigators (C. C. and W. R.) identified articles eligible for further review by performing an initial screening of identified title and abstract. We used broad inclusion criteria for studies during this step, which could be included by either of the two investigators. Further screening was based on full-text review. The results of two reviewers were compared, and disagreements were resolved by discussion and consensus. The first author's surname and the year of publication of the article were used for the identification purpose. We extracted data from studies concerning study design, baseline patient characteristics, categories of BMI, follow-up, sample size, and number of events. We also extracted the hazard ratios (HRs) of time-to-event data, as suggested by Parmar and colleagues, directly from the original study, or by reading off survival curves to estimate the log HR and its variance [12]. When studies reported mortality at several time intervals, we selected the longest follow-up period for analysis.

### Statistical Analysis

We extracted dichotomous data form all studies reporting number of patients with events and total participants and pooled them to calculate relative risks (risk ratios, RRs) with 95% confidence interval (CI). We further performed a baseline risk-adjusted analysis of survival data to determine if the main results were robust when quantitative pooling was limited to those studies in which we could calculate pooled adjusted all-cause mortality HR. We used the statistic of *I^2^* to efficiently test for the heterogeneity, with *I^2^*<25%, 25–75% and >75% to represent low, moderate and high degree of inconsistency, respectively [13]. Statistical heterogeneity was defined as an *I^2^* statistic value of more than 50% [13]. In analyses, if the heterogeneity was low then we used a fixed-effect model, or else applied the random-effect model [14]. Furthermore, we examined the variables concerning age, lung function, study population (hospital-based or community-based), and length of follow-up in a meta-regression model to explore for possible heterogeneity. We performed a sensitivity analysis, in which a study was removed at a time while the rest was analyzed, to evaluate whether the results could have markedly been affected by that single study [14]. We used Egger's linear regression test to find a potential publication bias [15]. All analyses were performed with Review Manager (version 5.1, The Cochrane Collaboration, Oxford, UK) and Stata (Version10.0, Stata Corporation, College Station, TX, USA). A 2-tailed *P* value of less than 0.05 was judged as statistically significant.

## Results

**Figure 1:**
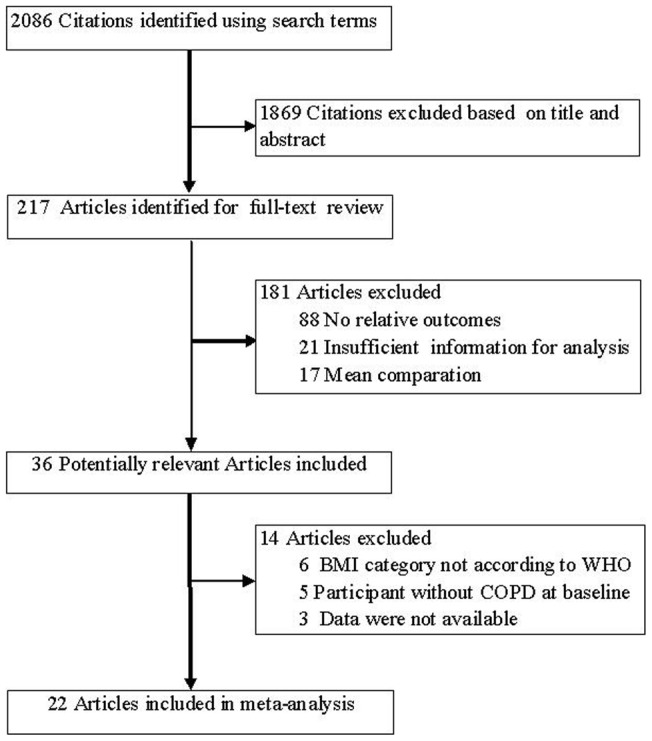
Flow Diagram of the Literature Search and Trial Selection Process.

The electronic database search identified 2086 citations. Of these, the first screening excluded 1869 citations based on abstracts or titles, leaving 217 articles for full-text review. The excluded 181 studies had no relative outcomes, insufficient information, and means or standard deviations comparison for BMI. On more detailed review, an additional 14 papers were excluded for the following reasons: BMI classification was not according to WHO [16]–[21]; participant was without COPD at baseline [22]–[26]; or data was unavailable [27]–[29]. We finally included 22 studies in our systematic review and meta-analysis [30]–[51]. The detailed steps of our literature search are shown in Figure 1.

**Table 1:** Characteristics of Studies Included in the Meta-analysis.

Source	No. of Participants	Study Design	FEV1,%, Predicted, Mean (SD), [Range]	Age at Follow-up, Mean(SD)[Range], y	BMI categories reported (kg/m2)	Study Quality^*^	Follow-up, y
Chang et al,^30^ 2007	84	Retrospective review	36 [14–83]	69.5 (12)	Underweight <20, normal 20–25, overweight 26–30, obese >30	6	1
Ringbaek et al,^31^ 2004	221	Prospective cohort	29.8 (10.7)	NA	Underweight <20, normal 20–24.9, overweight ≥25	7	4.9
Esteban et al,^32^ 2010	543	Prospective longitudinal	55.0±13.3	68.3±8.3	Underweight ≤21, normal/overweight >21	6	3
Seersholm,^33^ 1997	342	Prospective cohort	52.1 (29.5)	45.5 (10.7)	Underweight <20, normal/overweight ≥20	7	7.6
Marti et al,^34^ 2006	128	Retrospective review	25.4 (8.8)	68.9 (9.7)	Underweight <20, normal 20–24.9, overweight 25–29.9, obese ≥30	7	3
Pothirat et al,^35^ 2007	195	Prospective cohort	41.97 (15.45)	67.7 (7.8 ) [48–86]	Underweight<18.5, normal 18.5–25, overweight 25–30, obese ≥30	6	3.8
Gunen et al,^36^ 2005	205	Prospective cohort	62.7 (21.2)	64.8 (9.3)	Underweight <20, normal 20–24.9, overweight ≥25	7	3
Collins et al,^37^ 2010	424	Retrospective review	NA	NA	Underweight <20, normal 20–24.9, overweight 25–29.9, obese ≥30	NA	1
Landbo et al,^38^ 1999	2132	Prospective	Man: 64.7 (18.1) Woman: 66.1 (16.6)	Man:57.7 (11.0) Woman:55.0 (10.8)	Underweight <20, normal 20–24.9, overweight 25–29.9, obese ≥30	8	17
Budweiser et al,^39^ 2007	188	Prospective cohort	31.0±9.6	64.5 (8.0)	Underweight/low-normal <25, overweight ≥25	7	5
Hallin et al,^40^ 2007	261	Prospective cohort	NA	NA	Underweight <20, normal 20–25, overweight 25–30, obese >30	6	2
Chailleux et al,^41^ 2003	4088	Prospective cohort	Man: 68 (9) Woman:70 (10)	Man: 31 (12) Woman: 34 (11)	Underweight <20, normal 20–24, overweight 25–29, obese ≥30	8	5
Hansen et al,^42^ 2001	1095	Retrospective cohort	NA	61.8 (9.9)	Underweight <20, normal 20–24.9, overweight 25–30, obese ≥30	6	8.9
Jordan et al,^43^ 2010	2439	Retrospective cohort	NA	64.28	Underweight<18.5, normal 18.5–24.9, overweight 25–29.9, obese ≥30	6	10
Machado et al,^44^ 2006	435	Prospective cohort	31.4 (8.0)	66.6 (7.6)	Underweight <18.5, normal 18.5–24.9, overweight 25–29.9, obese ≥30	7	7
Almagro et al,^45^ 2009	316	Prospective cohort	NA	72.1 (9.6)	Underweight <20, normal 20–25, overweight 25–30, obese >30	7	3
Haruna et al,^46^ 2010	251	Prospective cohort	50.3 (17.0) [10.0–95.1]	68.7 (7.0) [47–88]	Underweight <18.8, normal/overweight ≥18.8	7	8
Ringbaek et al,^47^ 2005	869	Prospective cohort	Man: 43.3 (17.4) Woman: 45.2 (15.8)	Man: 57.7 (8.5) Woman: 55.0 (6.9)	Underweight <20, normal 20–24.9, overweight 25–29.9, obese ≥30	7	13.3
Schembri et al,^48^ 2009	3343	Prospective cohort	NA	NA	Underweight <18.5, normal 18.5–24.9, overweight 25–29.9, obese ≥30	6	1.9
Prescott et al,^49^ 2002	1612	Prospective cohort	Man: 66.3 (20.6) Woman: 67.3 (21.7)	Man: 56.6 (9.7) Woman: 55.8 (9.0)	Underweight <18.5, normal 18.5–24.9, overweight 25–29.9, obese ≥30	8	14
Vestbo et al,^50^ 2006	1898	Epidemiologic study	NA	NA	Underweight <18.5, normal 18.5–24.9, overweight 25–29.9, obese ≥30	7	7
Tsimogianni et al,^51^ 2009	81	Retrospective cohort	47 (17) [15–87]	68 (9) [44–87]	Underweight/normal <25, Overweight/obese ≥25	6	3

Abbreviation: BMI: body mass index; NA, data not available. ^*^Study quality was judged based on the Newcastle-Ottawa Scale (range, 1–9 stars).

The characteristics of 22 included studies are summarized in Table 1. A total of 21,150 participants with COPD were included in these studies. The selected studies were published between 1997 and 2010. Median duration of follow-up ranged from 1 to 17 years, with 11 of the 22 studies had more than 5 years of follow-up. All studies reported the mortality in COPD patients according to BMI category. 10 studies were community based [31], [33], [36], [38], [41]–[43], [48]–[50]. Adjusted data were available for 16 studies [31], [33], [34], [38]–[51], either as reported or by reading off survival curves. Risk measures concerning age, gender, FEV_1_%pred, and smoking were frequently adjusted. Analyses were also adjusted for other risk factors in some studies, such as outdoor activity, inhalation, PaO2, PaCO2, cor pulmonale, and COPD-related hospitalization. One study reported HRs of mortality in man and woman separately [38], and each group was considered as a separate study in analyses.

**Figure 2:**
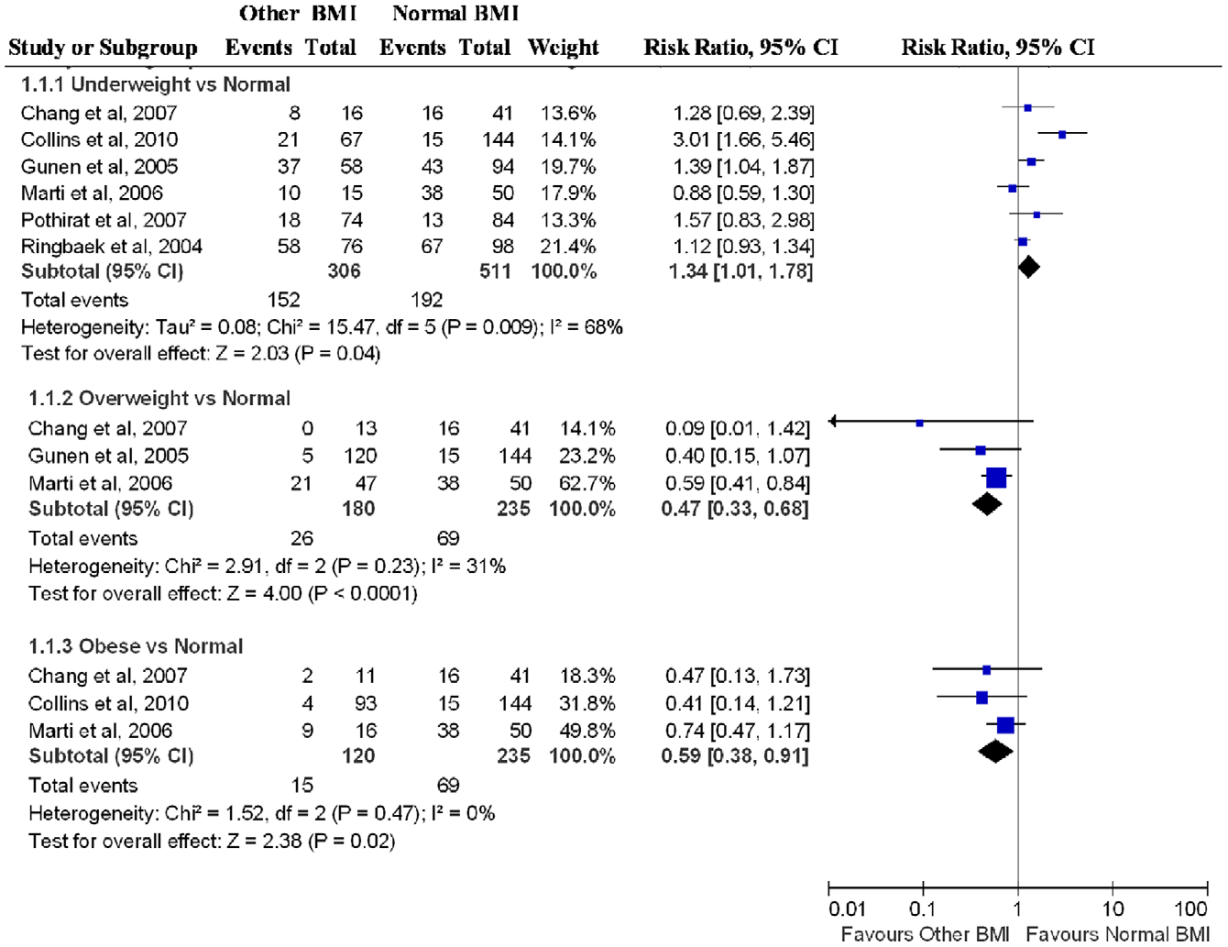
Relative risks of Mortality with Body Mass Index among Patients with Chronic Obstruct Pulmonary Disease.

Dichotomous clinical outcomes are reported as RR and survival data as HR. We pooled RRs of deaths for different levels of BMI compared with a reference of normal BMI of 18.5 kg/m^2^ to 24.9 kg/m^2^. Compared with patients with normal BMI, underweight individuals were associated with a higher mortality (RR  = 1.34, 95% CI  = 1.01–1.78), whereas overweight (RR  = 0.47, 95% CI  = 0.33–0.68) and obese (RR  = 0.59, 95% CI  = 0.38–0.91) patients were associated with a lower mortality (Figure 2). The RR for mortality were significantly higher in underweight individuals compared with individuals with BMI >20 kg/m^2^ (RR  = 1.65, 95% CI  = 1.29–2.11; Table 2). We also found significant increased risk of mortality in patients having a BMI of 24.9 kg/m^2^ or less compared with BMI ≥25 kg/m^2^ (RR  = 3.02, 95% CI  = 2.02–4.52).

**Table 2:** Body Mass Index and Mortality in Chronic Obstruct Pulmonary Disease.

	Unadjusted outcomes						Risk-adjusted outcomes					
Body Mass Index (kg/m^2^)	No.^a^	Subjects	RR [95% CI]	*P*	*I^2^*,%	*P^b^*	No.^a^	Subjects	HR [95% CI]	*P*	*I^2^*,%	*P^b^*
<18.5 vs. 18.5–24.9	6	1,161	1.34[1.01,1.78]	0.04	68	0.009	8	14,163	1.48[1.26,1.75]	<0.00001	66	0.005
25–29.9 vs. 18.5–24.9	3	510	0.47[0.33,0.68]	<0.0001	31	0.23	8	12,659	0.78[0.65,0.94]	0.008	79	<0.0001
≧30 vs. 18.5–24.9	3	439	0.59[0.38,0.91]	0.02	0	0.47	8	14,365	0.69[0.54,0.89]	0.004	76	0.0001
<18.5 vs. ≧18.5	8	5,744	1.65[1.29,2.11]	<0.0001	83	<0.00001	2	593	1.53[124,1.88]	<0.0001	0	0.74
<24.9 vs. ≧25	5	1,452	3.02[2.02,4.52]	<0.00001	26	0.25	2	209	2.361.65,3.36]	<0.00001	0	0.63

a Number of studies; ^b^ *P* value of Q-test for heterogeneity test.

**Figure 3:**
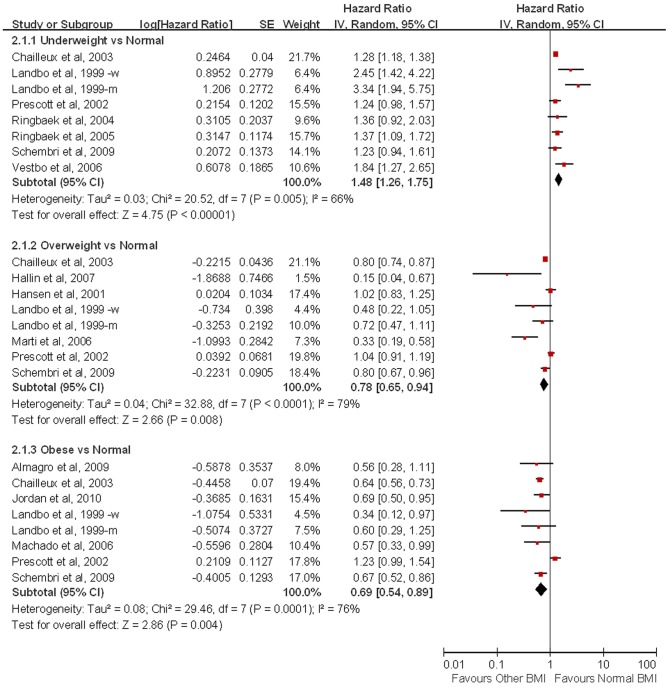
Hazard ratios of Mortality with Body Mass Index among Patients with Chronic Obstruct Pulmonary Disease (HR was adjusted for age, gender, FEV_1_%pred, smoking and so on. The HR of Chailleux et al. was extracted by reading off survival curves).

Sixteen studies analyzed by a Cox proportional hazards regression model to adjust for baseline differences and were included in a combined risk adjusted analysis. As shown in Figure 3, the HR of mortality in underweight patients was 1.48 (95% CI  = 1.26–1.75) when compared with those with a normal BMI. Compared with patients with normal BMI, overweight (HR  = 0.78, 95% CI  = 0.65–0.94) and obese (HR  = 0.69, 95% CI  = 0.54–0.89) patients were associated with lower mortality. In addition, underweight patients had higher mortality compared with those with BMI values of 20 kg/m^2^ or more (HR  = 1.53, 95% CI  = 1.24–1.88). Likewise, compared with individuals with a BMI ≥25 kg/m^2^, underweight and normal BMI patients were associated with higher mortality (HR  = 2.36, 95% CI  = 1.65–3.36). We further performed a subgroup analysis by different BMI cut-off for underweight. The heterogeneity of underweight versus normal turned out to be insignificant (*P*_heterogeneity_ = 0.16, *I^2^* = 45%) in the studies with standardized BMI cut-off. In the subgroup analysis of obese versus normal, the heterogeneity was significantly decreased in the studies without standardized BMI cut-off (*P*_heterogeneity_ = 0.68, *I^2^* = 0%).

To explore the heterogeneity among studies of BMI and mortality, a sensitivity analysis was performed. In risk-adjusted analysis, when one study (group of man) was excluded [38], the heterogeneity of underweight versus normal turned out to be insignificant (*P*_heterogeneity_ = 0.16, *I^2^* = 35%), with an HR of 1.36 (95% CI, 1.21–1.52), whereas exclusion of any other studies did not influence the results. A study by Prescott et al. was the main origin of heterogeneity in the comparison of obese versus normal [49]. After exclusion of this study, the heterogeneity was significantly decreased (*P*_heterogeneity_ = 0.91, *I^2^* = 0%; HR  = 0.64, 95% CI  = 0.58–0.71). However, pooled HR of mortality in comparison of overweight versus normal was not excessively influenced by any single study. As to unadjusted data analysis, similar procedures on sensitivity analysis were performed. After exclusion of one study [37] about the comparison between underweight and normal, the heterogeneity was significantly decreased (*P*_heterogeneity_ = 0.30, *I^2^* = 18%; RR  = 1.21, 95% CI  = 1.04–1.40). The pooled result was not affected by any single study in comparison of underweight versus BMI values of 18.5 kg/m^2^ or more. We further performed meta-regression to identify the sources of heterogeneity. We evaluated the source of heterogeneity by age, lung function, study population, and length of follow-up. We found that study population were a significant source of heterogeneity in the comparison of underweight versus BMI values of 18.5 kg/m2 or more (tau^2^ = 0.0132, *P* = 0.001). However, meta-regression did not indicate age, lung function, study population, or length of follow-up could explain the significance between-study heterogeneity in the comparison of underweight versus normal.

### Publication bias

We used Egger's regression asymmetry test to access the publication bias of literatures. The results showed no evidence of publication bias (t = 0.44, *P* = 0.684 for underweight versus normal; t = −6.76, *P* = 0.094 for overweight versus normal; and t = −0.87, *P* = 0.543 for obese versus normal).

## Discussion

To our knowledge, this study is the first comprehensive meta-analysis to date that has assessed the relationship between BMI and mortality in COPD patients. The findings from our study indicated that reduced BMI was associated with an increased risk of mortality in COPD. A higher risk of death was found not only in underweight patients, but also in those with normal BMI.

In our study, we found higher mortality associated with underweight individuals among patients with COPD when compared with those having a normal BMI value, while lower overall mortality in overweight and obese patients. Statistically similar results were obtained after risk adjustment analysis. These results demonstrated an inverse relationship between BMI and mortality among patients with COPD, which corresponded with most of the related studies. However, there was evidence of heterogeneity between studies in our analysis. After sensitivity analysis by sequential omission of individual studies, heterogeneity was effectively decreased or removed from all but two comparisons. In our study, the reasons for heterogeneity were unclear. It might have been due to methodological differences among studies, particularly on the cutoff point for underweight patients.

One of the most important finding in this study was that individuals even with a normal BMI had a higher risk of mortality. In recent years, lots of studies combined several variables including BMI to predict mortality in COPD. A good example is the BODE-index (**B**ody mass index, **O**bstruction, **D**yspnea, **E**xercise capacity) [4], a multidimensional 10-point scale in which higher scores indicate a higher risk of death. For body-mass index the values were 0 or 1, and the inflection point of BMI was 21 kg/m^2^. However, our results showed that BMI category associated with lower risk of mortality was 25 kg/m^2^ or more. Thus, a more reasonable scoring system concerning BMI might be a better predictor of mortality among patients with COPD.

The association between low BMI and poor survival among patients with COPD could be several reasons such as diaphragmatic muscle weakness [50], decreased lung function [52], and systemic inflammation [53], all of which is related to weight loss. Furthermore, proinflammatory status reflected by acute-phase proteins, tumor necrosis factor-a receptors, and soluble adhesion molecules is related to increasing resting energy expenditure in patients with COPD [54]. However, loss of skeletal muscle mass is the main cause of weight loss in COPD, whereas loss of fat mass contributes to a lesser extent [55]. Published data showed that fat-free mass index (FFMI) provided information in addition to BMI and assessment of FFMI was also suggested in the routine assessment [56].

Usually, obesity is a recognized risk factor for insulin resistance, obstructive sleep apnea, and cardiovascular disease. However, our study found that for patients with COPD being overweight or obese had a protective effect against mortality. The pathophysiological basis for this apparent obesity paradox is unknown. A recent study found thin patients with COPD to have more cardiovascular complications and a higher mortality rate [57]. In addition, Landbo et al thought that obesity in itself contributes to low FEV_1_, leading to those subjects being classified as having severe COPD while in fact they may not have experienced severe disease or a severe decline in lung function [38]. However, we could not obtain enough statistical power to explore those hypotheses because there were no sufficient data supplied in published studies. Further studies are needed to detect it by various methods, such as adjustment of obstructive ratio, FVC or subgroup analysis of individuals with severe flow limitation.

Considering deleterious effects of malnutrition in COPD patients, studies have been conducted and focused on the efficacy of nutritional supplementation therapy. Planas et al found administration of nutritional supplements to have a significant improvement in body weight and handgrip strength, decrement in airflow limitation, and increment in quality of life [58]. Raherison and Girodet thought that achieving an optimal nutritional status could delay the progression of COPD and might also reduce the risk of early mortality [59]. However, nutritional supplementation was only suggested as a part of the treatment approach in some specific individuals [60], [61]. Therefore, changing the BMI by nutritional supplementation, which improved COPD outcomes, needed further investigation.

As a systematic review, our findings and interpretations were limited by the quality and quantity of included studies. Firstly, the cutoff point for underweight was in disparity among studies and might have led to an underestimate risk of mortality in patients with low BMI. Secondly, detailed information, such as respiratory mortality and sex difference in mortality, were unavailable in most studies, which limited our further assessment of those confounding factors at the patient level, and were incorporated into the analysis. Thirdly, only studies written in English were included in this meta-analysis, which might have led to the bias. Lastly, as a meta-analysis, we were unable to get enough data to assess the relationship between BMI and mortality in different classes of obesity. There may be a ceiling BMI that is associated with better prognosis and this is not explored in the current study. Further studies should be more informative and then subgroup analysis by degree of obesity would be available.

In conclusion, our systematic review of 22 studies comprising 21,150 subjects showed that for patients with COPD being overweight or obese had a protective effect against mortality. However, the importance and efficacy of nutritional management of patients with COPD needed further clarification in well-designed, adequately powered clinical studies.
